# Real-time metabolic monitoring under exhaustive exercise and evaluation of ventilatory threshold by breathomics: Independent validation of evidence and advances

**DOI:** 10.3389/fphys.2022.946401

**Published:** 2022-08-12

**Authors:** Giovanni Pugliese, Phillip Trefz, Matthias Weippert, Johannes Pollex, Sven Bruhn, Jochen K. Schubert, Wolfram Miekisch, Pritam Sukul

**Affiliations:** ^1^ Department of Anesthesiology and Intensive Care Medicine, Rostock Medical Breath Research Analytics and Technologies (ROMBAT), Rostock University Medical Centre, Rostock, Germany; ^2^ Department of Atmospheric Chemistry, Max Planck Institute for Chemistry, Mainz, Germany; ^3^ Institute of Sport Science, University of Rostock, Rostock, Germany

**Keywords:** anaerobic threshold, volatile organic comound, proton transfer reaction time-of-flight mass spectrometry, non-invasive monitoring, lactate threshold, ventilatory threshold

## Abstract

Breath analysis was coupled with ergo-spirometry for non-invasive profiling of physio-metabolic status under exhaustive exercise. Real-time mass-spectrometry based continuous analysis of exhaled metabolites along with breath-resolved spirometry and heart rate monitoring were executed while 14 healthy adults performed ergometric ramp exercise protocol until exhaustion. Arterial blood lactate level was analyzed at defined time points. Respiratory-cardiac parameters and exhalation of several blood-borne volatiles changed continuously with the course of exercise and increasing workloads. Exhaled volatiles mirrored ventilatory and/or hemodynamic effects and depended on the origin and/or physicochemical properties of the substances. At the maximum workload, endogenous isoprene, methanethiol, dimethylsulfide, acetaldehyde, butanal, butyric acid and acetone concentrations decreased significantly by 74, 25, 35, 46, 21, 2 and 2%, respectively. Observed trends in exogenous cyclohexadiene and acetonitrile mimicked isoprene profile due to their similar solubility and volatility. Assignment of anaerobic threshold was possible via breath acetone. Breathomics enabled instant profiling of physio-metabolic effects and anaerobic thresholds during exercise. Profiles of exhaled volatiles indicated effects from muscular vasoconstriction, compartmental distribution of perfusion, extra-alveolar gas-exchange and energy homeostasis. Sulfur containing compounds and butyric acid turned out to be interesting for investigations of combined diet and exercise programs. Reproducible metabolic breath patterns have enhanced scopes of breathomics in sports science/medicine.

## Introduction

The analysis of volatile organic compounds (VOCs) in human breath offers vast possibilities for the development of non-invasive diagnostic techniques (Miekisch et al., 2004). Breath biomarkers could provide new and unique insights into metabolic (Sukul et al., 2018; 2022b), physiological (King et al., 2010b; Sukul et al., 2016, 2022a) or pathophysiological processes (Nakhleh et al., 2017; Trefz et al., 2019; Löser et al., 2020). VOCs are exhaled shortly after their production and may therefore deliver systemic information quickly and non-invasively. Despite the short time between cellular release of volatiles and their exhalation, real-time monitoring of metabolic processes has only been explored in a rudimentary fashion so far. Today, research is increasingly directed towards the detection of concentration changes rather than the detection of unique biomarkers (Sukul et al., 2016; Pugliese et al., 2019; Löser et al., 2020).

In recent years, the dependency of volatile breath markers on physical stress could be demonstrated (Ament et al., 1999; Karl et al., 2001; King et al., 2009, 2010b) as well as the dependency of breath VOC concentrations on general physiological effects (Sukul et al., 2014, 2015, 2016; 2017b; 2017a). It could also be shown, that concentrations of specific exhaled volatiles depend on physiological parameters such as cardiac output or minute ventilation (Pabst et al., 2007; King et al., 2010b). Minute or pronounced changes in respiratory and/or hemodynamic parameters are immediately reflected on such volatiles. For instance, endogenous isoprene exhalation increases with muscle activity, -perfusion and -washout (King et al., 2010a) whereas, decrease in its concentrations denotes the performance (%) of the conventional forced expiratory volume (FEV) maneuver (Sukul et al., 2016). Given the fact that the actual *in vivo* origin of such important VOCs is still uncertain (Sukul et al., 2021), observed changes are rather hard to assign certainly upon specific physiological and/or metabolic effect(s), under any given condition. A previous study showed that exhaled acetone concentrations may be used to determine anaerobic threshold (AT) (Schubert et al., 2012), while another study described how prolonged exercise is reflected in breath VOC concentrations (Henderson et al., 2021). Further, short term metabolic effects of dietary interventions on breath VOCs (Spacek et al., 2015; Hageman et al., 2019; Pugliese et al., 2019) and the potential for assessment of metabolic adaptation with respect to metabolic disorders could be demonstrated (Trefz et al., 2019).

Monitoring metabolic and physiologic processes during exercise is interesting from several points of view. Personalized assessment of metabolic adaptation could enable optimized training for professional athletes, but also for amateur or private sport or with respect to therapeutic exercise interventions. Breath VOCs may deliver real-time information on energy consumption or production in addition to established methods like lactate analysis or spirometry. Considering the available knowledge of exercise physiology and metabolism, adding VOC profiling under various experimental conditions may lead us closer to the true endogenous origin(s), kinetics and dynamics of exhaled biomarkers.

While AT denominates a physiological breakpoint associated with aerobic capacity/performance, lactate threshold (LT) and ventilatory threshold (VT) are used as methodological determinants of AT. A stepwise exercise protocol i.e. suitable for determination of the lactate threshold (LT) was applied in the above-described study to assess AT by means of breath VOCs (Meyer et al., 2005). Here we followed a ramp exercise protocol i.e. better suited for the determination of VT in order to assess AT via VOC exhalation. A major limitation of the aforementioned study was the constraint to only three breath VOCs due to the use of a proton-transfer-reaction-mass-spectrometer (PTR-MS) equipped with a quadrupole MS. This limitation was lifted in our study since we applied a PTR coupled with a time-of-flight-mass-spectrometer (ToF-MS) which allows the simultaneous monitoring of hundreds of exhaled VOCs. The following research questions were addressed:

• Can the VOC based AT determination as suggested by Schubert et al. (Schubert et al., 2012) be applied in a ramp exercise protocol?
• Which endogenous compounds in exhaled breath show significant changes during exhaustive exercise and can these changes be attributed to metabolic processes?
• Are there correlations between breath VOCs and spirometric parameters such as minute ventilation or oxygen consumption?

## Materials and methods

### Study design

Sample size was calculated by analysis of variance (ANOVA) test. Minimal detectable differences (MDDs) in mean substance intensities of 350 ncps (i.e., protonated ion counts, normalized onto PTR primary ion counts) along with an expected residual standard deviation of 200 were applied (as per our experiences from previous clinical studies) for the determination of < 5% differences in exhaled VOCs at the low parts per trillion by volume (pptV) range. An alpha value of 0.05 at the test power of 0.95 was attained, while considering a minimum sample size of 10. Thus, 14 healthy participants were enrolled in the study. All included subjects were young to mid-aged adults with normal lifestyle (residents of Rostock, Germany) and without any acute or chronic disease/condition/therapy. They were not undertaking any special diet/nutritional supplement and were without the habits of smoking cigarette and/or drinking alcohol. Laboratory ambient conditions were constant throughout the experiments (temperature: between 19–21°C and humidity: 30%). Ambient/inspiratory air was measured continuously in parallel to determine any immediate confounding effects from the laboratory environment. The study was approved by the institutional ethics committee (Approval No. A 2011 67) in accordance with the amended Declaration of Helsinki guidelines. All subjects gave written and informed consent prior to participation. Table 1 gives an overview over the study population.

**TABLE 1:** Study population data (median and range).

Age [years]	26.5 (18–53)
Sex (male; female) [n]	8m; 6f
Body Height [cm}	174.5 (155–197)
Bod Weight [kg]	71 (48–103)
BMI [kg/m^2^]	22.8 (19.7–27.1)

### Incremental exercise protocol

Participants performed a standard ramp-like protocol (25 W/min), leading to exhaustion within ca. 10 min in active, healthy subjects. Such protocol is recommended to elicit VO_2_ peak as well as enable the determination of ventilatory thresholds (Buchfuhrer et al., 1983). After a standardized active warm-up of five minutes at 50 W, all participants performed an incremental cycling test on an electromagnetically braked bicycle ergometer (SRP 3000, Sportplus Germany) until exhaustion, starting at a power output of 50 W with a stepwise increase of 25 W/min. The incremental test was stopped if cycling cadence dropped below 60 rpm and participants were no longer able to maintain the required power output despite strong verbal encouragement.

### Experimental setup and instrumentation

**FIGURE 1:**
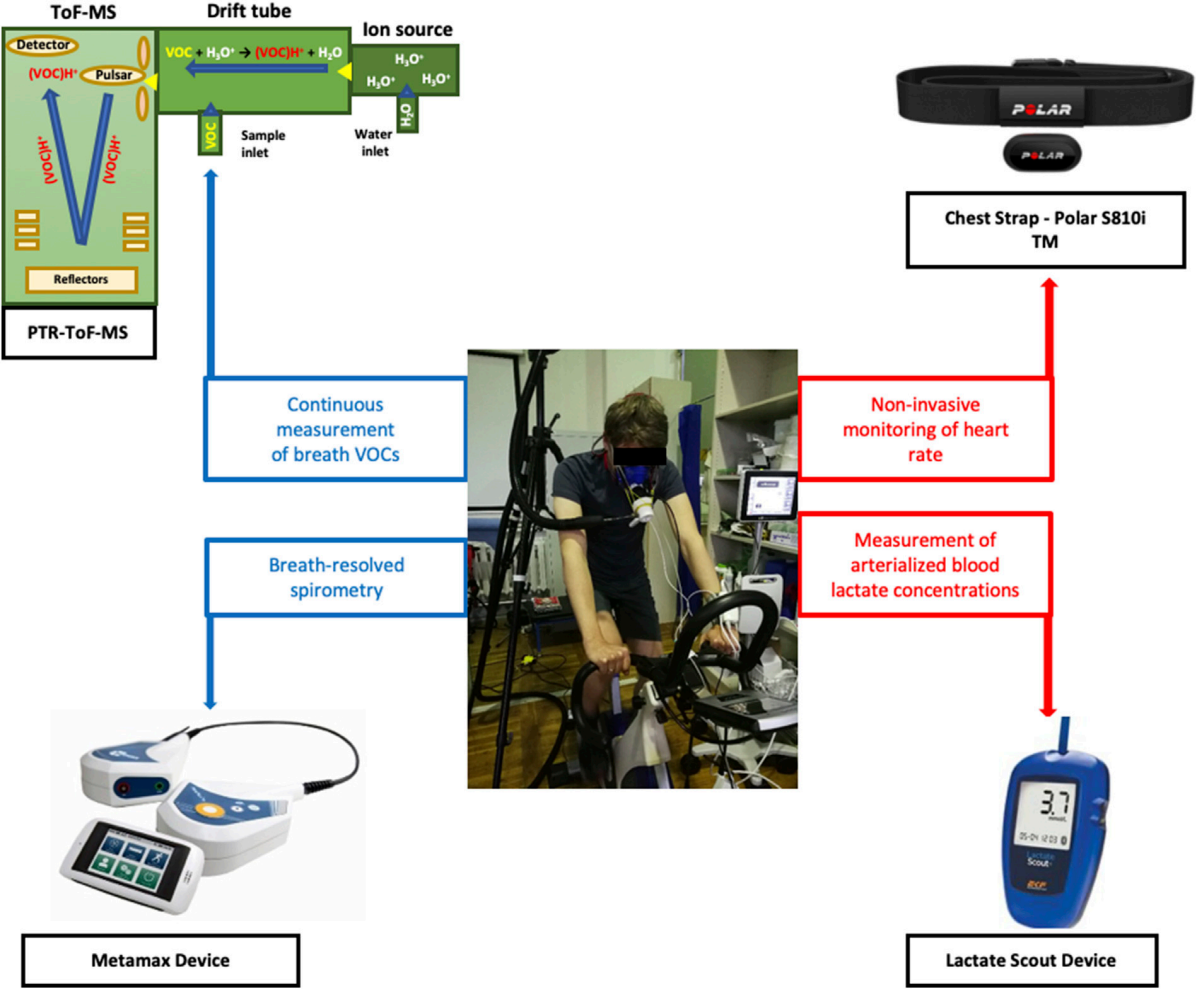
Overview of the experimental setup and instruments. A test subject on the cycle-ergometer along with four analytical instruments are presented.

Four analytical instruments were synchronized to measure several respiratory and hemodynamic parameters (Figure 1). Breath VOCs were measured continuously via PTR-ToF-MS and breath-resolved ventilatory changes were monitored via flow-volume spirometry. Heart rate was measured via a chest strap sensor and blood lactate concentrations were measured via electrochemical analysis of arterialized blood.

### Spirometric analysis

A commercially available face mask (CareFusion, Höchberg, Germany) was used during the experiments. Spirometry was done via metamax device (Cortex Biophysik GmbH, Leipzig, Germany). Volume of oxygen consumption (VO_2_), volume of CO_2_ production (VCO_2_), minute ventilation (VE), tidal volume (TV), respiratory rate (RR) and respiratory gas exchange ratio (RER) were recorded in a breath-by-breath manner via an electrochemical sensor. Data were sampled at the rate of at 0.2 Hz and transmitted telemetrically. A daily calibration of the measurement system was carried out.

### Blood lactate analysis

Capillary blood for serum lactate analysis was taken from the earlobes. 10 µl blood were used for this purpose. Arterialization of the blood was done by treating the earlobe with a perfusion enhancing ointment (Finalgon, Thomae, Biberach, Germany). Lactate analysis was performed at the beginning of the excercise during warm up and then every 2 minutes after the start of the ramp protocol. Lactate concentrations were determined electrochemically by LactateScout (Senslab, Germany).

### Measurement of heart rate

Heart rate was monitored by means of a HR monitor (Polar RS800, Finland) consisting of a transmitter built as a chest strap and a watch used as a receiver. HR was recorded at 0.2 Hz.

### Breath analysis

PTR-ToF-MS breath sampling, measurement and data analysis have been described before (Trefz et al., 2013; Sukul et al., 2014). Briefly, breath was sampled continuously in side-stream mode by means of a heated 6 m silcosteel transfer line while the participant was breathing through a sterile face mask. The mask was adapted for continuous PTR sampling via a Teflon piece with a side-stream Luer connection where the PTR transfer line was connected. No breathing resistance was introduced through the mask and Teflon piece. A PTR-ToF-MS 1000 (Ionicon Analytik GmbH, Innsbruck, Austria) was used in the study.

The PTR settings were as follows. The time resolution was 200 ms and the sampling flow was 20 sccm. The drift voltage was 610 V, the drift temperature was 75°C and the drift tube pressure was 2.3 mbar, resulting in an E/N ratio of 138 Td. Mass scale was recalibrated after every run of 60 s. Masses used for that purpose were 21.02 (H_3_O^+^-Isotope), 29.99 (NO^+^), 59.05 (protonated acetone). Processing of data was done *via* PTR-MS Viewer 3 (Ionicon Analytik GmbH, Innsbruck, Austria) and expiratory and inspiratory phases were recognized by means of a MATLAB based algorithm (“breath tracker”). The function of the algorithm has been described before (Trefz et al., 2013). Briefly, an endogenous compound that has relatively higher concentration in expiration compared to the inspiration is used as a tracker mass to differentiate between expired and inspired phases. Acetone was used for this purpose. Phase resolution determined by means of the algorithm is then applied to all m/z of interest.

Alveolar data was averaged over time intervals of 60s resulting in one mean value for each increment of the ramp protocol. Exercise levels were then presented as percentage exercise levels with respect to the maximum power to unify the varying individual training status.

VOCs were quantified by performing calibrations with pure reference substances. A liquid calibration unit (LCU, Ionicon Analytic, Innsbruck, Austria) was used to generate VOC standards at specific concentrations and adapted sample humidity from either liquid standard solutions or standard gas cylinders (Trefz et al., 2018). Two standard gas cylinders were used, one included n–C_1_ to C_10_ aldehydes, 2-propenal, and 2-butenal; and the second contained formaldehyde, acetaldehyde, methanol, ethanol, isoprene, acetone, 2-propenal, acetonitrile, 2-butanone, benzene, 2-butenal, toluene, chlorobenzene, o-xylene, and 1,2-dichlorobenzene. In addition, gas standards of isopropanol, dimethylsulfide, methyl acetate and ethyl acetate were generated from liquid standard solutions. The concentrations for compounds not included in the standard were calculated using reaction rates and transmission curves (Lindinger et al., 1998).

### Statistical analysis

For each participant, breath VOC concentrations and spiroergometric parameters determined at each workload level were selected to calculate changes from the last minute of warm-up (WU2). Statistically significant differences from the WU2 were determined by means of Friedman repeated measures analysis of variance (ANOVA), where Student-Newman-Keuls test was used for post-hoc comparisons to determine the origin of difference identified by the ANOVA. A *p*-value < 0.05 represents significant difference. Linear correlation between VOCs and spiroergometric parameters were evaluated via Pearson product moment correlation analysis. Sigma Plot 14 (Systat Software GmbH, Erkrath, Germany) and SPSS 17 (IBM Software, Armonk, United States) were used for statistical analysis.

### Determination of the lactate threshold

The LT was defined at a lactate concentration of 1.5 mmol/L above the lowest value of the ratio lactate/performance (lactate-equivalent) which describes the onset of lactate increase. However, it is important to note that a ramp test is not ideally suited for the determination of LT (Meyer et al., 2005).

### Determination of the respiratory compensation point, ventilatory threshold and respiratory gas exchange ratio

The RCP and VT were calculated according to the V-slope method (Beaver et al., 1986). In addition, RER was calculated as VCO_2_/VO_2_.

### Determination of breath acetone anaerobic threshold

The AT was calculated from acetone according to the method of Schubert et al. (Schubert et al., 2012). Briefly, exhaled acetone concentrations are modeled as a function of time of exercise *via* 3rd order polynomial regression and the maximum of the regression curve is determined from the first derivate. This maximum corresponds to the AT.

## Results

Table 2 represents an overview over the breath VOCs and respiratory data.

**TABLE 2:** Heatmap based on normalized mean data from all 14 volunteers.

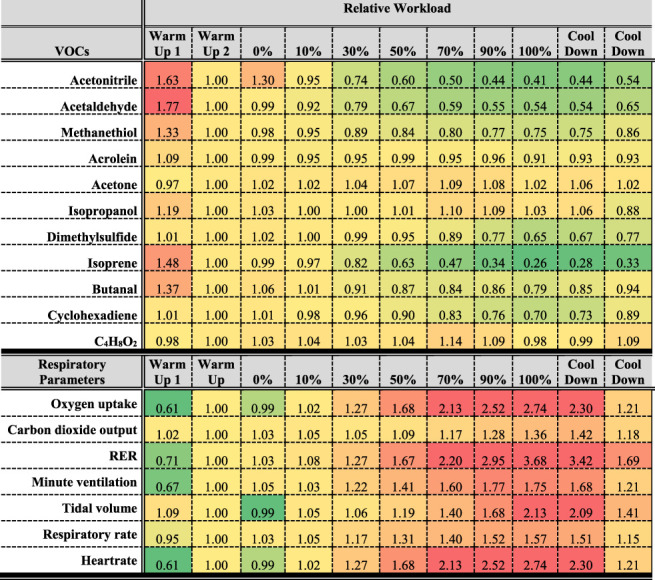

VOC and respiratory parameters were normalized to the last “warm up” measurement before starting the ramp protocol. Data was color coded to show relative differences, with red representing relatively high values (above warm up value) and green representing relatively low values (below warm up value).

### Respiratory gas exchange

**FIGURE 2:**
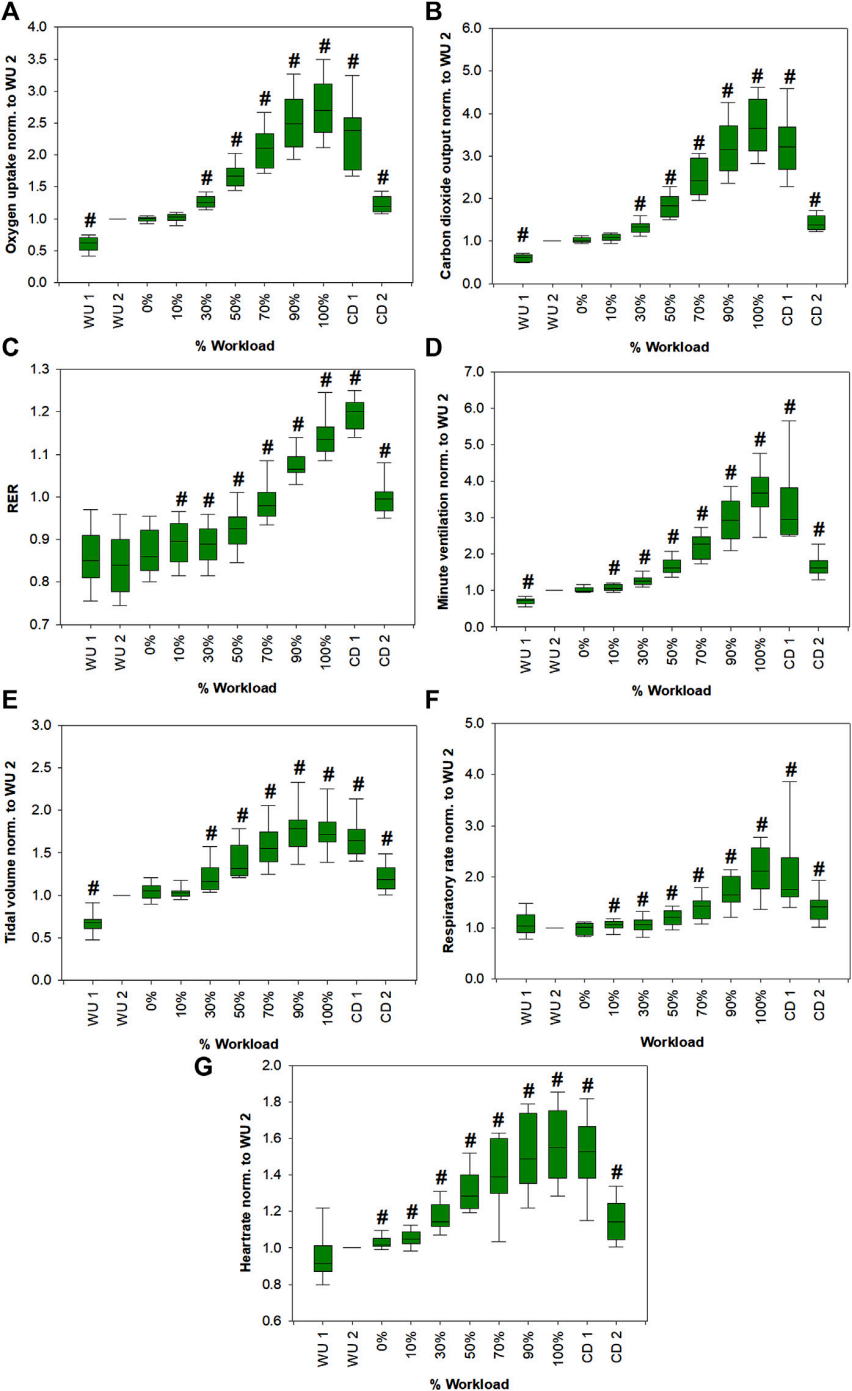
Changes in VO2 **(A)**, VCO2 **(B)**, RER **(C)**, VE **(D)**, TV **(E)**, RR **(F)** and HR **(G)** from all volunteers as a function of the relative workload. Except for RER, absolute values of all parameters were normalized to respective values at the last minute of warm-up (WU 2) for emphasis of relative changes. Statistically significant changes (Friedman repeated measures ANOVA on ranks, Student-Newman-Keuls post-hoc test, *p* < 0.05; except for TV. Due to normal distribution, a one-way repeated measures ANOVA was performed, Student-Newman-Keuls post-hoc test, *p* < 0.05) compared to WU 2 are marked with a hash. Corresponding *p*-values are shown in Supplementary Table S2.

Figure 2 shows changes of spirometric parameters from all volunteers as function of relative workload. Apart from RER, absolute values of all parameters were normalized to respective values at the last minute of warm-up (WU 2). RER (VCO_2_/VO_2_) value may vary from <0.7 to >1.2. WU 1 represents the first minute of warm up. VE, VO_2_, TV, RER as well as HR steadily increased from the beginning of exercise until the end of the workload and then they decreased during the cool down phase.

### Breath volatile organic compounds

**FIGURE 3:**
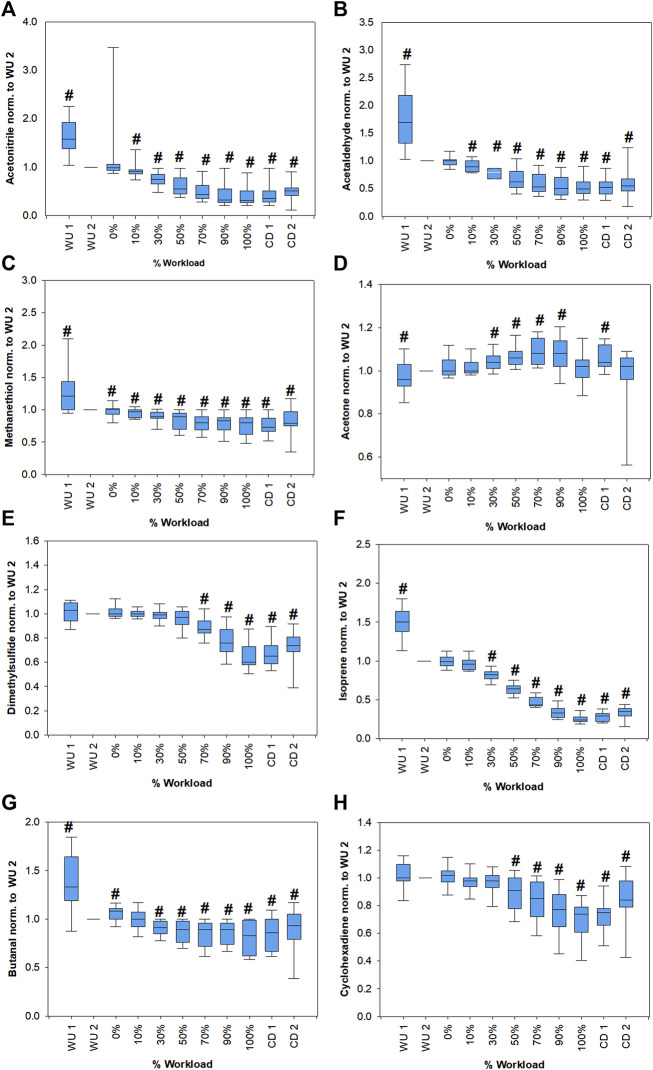
Changes of acetonitrile **(A)**, acetaldehyde **(B)**, methanethiol **(C)**, acetone **(D)**, dimethylsulfide **(E)**, isoprene **(F)**, butanal **(G)** and cyclohexadiene **(H)** concentrations from all volunteers as a function of the relative workload. Absolute concentrations of all compounds were normalized to respective values at the last minute of warm-up (WU 2) for emphasis of relative changes. Statistically significant changes (Friedman repeated measures ANOVA on ranks, Student-Newman-Keuls post-hoc test, *p* < 0.05) compared to WU 2 are marked with a hash. Corresponding *p*-values are shown in Supplementary Table S3.

Figure 3 shows changes in breath VOCs concentrations from all volunteers as function of relative workload. Only compounds that showed significant changes compared to WU 2 levels and clear trends are shown. Butanal, acetaldehyde, isoprene and methanethiol concentrations showed a significant increase at the begin of exercise followed by a significant decrease down to about 80, 55, 25 and 75%, respectively, of the WU 2 levels at the end of the workload. Then they increased during the cool down phase. Acetone showed an increase up to 90% workload then went back to WU 2 levels and increased again during cool down. Isopropanol did not show a clear trend, although normalized mean values shown in Table 2 suggested a comparable trend to acetone. C_4_H_8_O_2_ and acrolein are not shown since no significant changes could be observed. Box plots of acrolein, isopropanol and C_2_H_8_O_2_ can be found in Supplementary Figure S1. Statistical significance of changes in VOC concentrations within the first minute of warm up are also presented in Supplementary Table S1.

Linear correlation between VOCs and spirometric parameters as well as HR were evaluated via Pearson product moment correlation analysis. Results can be found in Table 3. Most VOCs showed statistically significant correlations, but the majority were weak correlations with correlation coefficients in the range of approximately 0.3–0.4. Only isoprene showed correlation coefficients >0.5 for all parameters except TV and acetaldehyde showed a correlation coefficient of 0.54 to heart rate. Isopropanol and C_4_H_8_O_2_ showed no significant correlations and acetone were only weakly associated with tidal volume and respiratory rate.

**TABLE 3:** Pearson product correlation coefficients between VOCs, spirometric parameters and HR.

VOCs	VO_2_	VCO_2_	HR	RER	VE	TV	RR
Acetonitrile	−0.27^**^	−0.27^**^	−0.45^**^	−0.21^**^	−0.30^**^	−0.15	−0.38^**^
Acetaldehyde	−0.45^**^	−0.40^**^	−**0.54** ^**^	−0.39^**^	−0.41^**^	−0.37^**^	−0.29^**^
Methanethiol	−0.36^**^	−0.34^**^	−0.30^**^	−0.25^**^	−0.30^**^	−0.34^**^	−0.10
Acrolein	−0.09	−0.13	−0.32^**^	−0.22^**^	−0.19	0.01	−0.27^**^
Acetone	0.14	0.08	−0.06	−0.136	−0.01	0.38^**^	−0.30^**^
Isopropanol	0.05	0.04	0.04	<0.01	0.02	0.16	−0.11
Dimethylsulfide	−0.25^**^	−0.27^**^	−0.27^**^	−0.26^**^	−0.23^**^	−0.28^**^	−0.02
Isoprene	−**0.55** ^**^	−**0.55** ^**^	−**0.56** ^**^	−**0.52** ^**^	−**0.58** ^**^	−0.25^**^	−**0.61** ^**^
Butanal	−0.34^**^	−0.35^**^	−0.35^**^	−0.40^**^	−0.38^**^	−0.11	−0.48^**^
Cyclohexadiene	−0.32^**^	−0.32^**^	−0.44^**^	−0.27^**^	−0.27^**^	−0.27^**^	−0.14
C_4_H_8_O_2_	−0.01	−0.01	−0.05	0.03	<−0.01	0.12	−0.13

^**^Correlation is significant at the 0.001 level. Correlation coefficients > 0.5 are marked in bold.

### Comparison between acetone anaerobic threshold with lactate threshold and ventilatory threshold

Acetone AT was quite comparable with both LT and VT with differences < 25% for most of participants (Table 4). The only exception was participant 1, for whom acetone AT was largely different from LT (48%) and VT (38%).

**TABLE 4:** Comparison between acetone AT with LT and VT

Volunteer	Acetone AT [W]	LT/[W]	VT [W]	Acetone AT/LT	Acetone AT/VT
1	130	250	208	0.52	0.63
2	200	200	190	1.00	1.05
3	177	n.a	191	n.a	0.93
4	203	n.a	247	n.a	0.82
5	211	200	206	1.06	1.02
6	212	275	212	0.77	1.00
7	184	225	208	0.82	0.88
8	213	200	239	1.07	0.89
9	174	175	166	0.99	1.05
10	182	n.a	155	n.a	1.17
11	265	275	n.a	0.96	n.a
12	231	200	265	1.16	0.87
13	264	n.a	273	n.a	0.97
14	162	n.a	135	n.a	1.20

n.a. denotes to “not applicable”.

## Discussion

We were able to monitor 11 VOCs with potential blood born origin (Figure 3, Supplementary Figure S1). We only included VOCs in our analysis that were expressed in higher amounts in exhaled breath compared to the room air. Still, several of these compounds cannot be clearly assigned as endogenous since they may have been taken up from the environment or present a mixed origin with contributions from endogenous as well as exogenous sources. Since individual performance varied between volunteers, a unification of the dataset was necessary. Therefore, the data is presented as percentage exercise levels with respect to the maximum power during exercise. This was necessary since training status differed between individuals. As expected, spirometric parameters increased significantly with increasing workload, reached a maximum at exhaustion (100% workload) and then started to decrease again during the cool down period (Figure 2).

### Endogenous compounds

As per previous observations, acetone exhalation is independent of respiratory flow and volume (Bikov et al., 2013; Sukul et al., 2017b). Acetone concentrations followed a similar trend as described by Schubert *et al.* (Schubert et al., 2012), i.e., acetone increases with the onset of exercise, reaches a maximum and then start to decrease (Figure 3D). Schubert et al*.* argued that the initial increase in exhaled acetone concentration can be explained by a mobilization of dextrose and beta-oxidation of fatty acids and when metabolism changes to anaerobic mode, it results in a decreased production of acetoacetate and thus leads to a decrease in acetone exhalation, which is further amplified through lactate infused inhibition of lipolysis. As a consequence, they suggested that exhaled acetone may be used to determine the AT. Their proposed method for determination of AT from the maximum of a 3^rd^ order polynomial fit of exhaled acetone concentrations were applied to our data and yielded a good agreement, with differences < 25%, of acetone AT and LT as well as the VT (Table 4). There was no clear tendency whether the anaerobic threshold determined from acetone closer resembles LT or VT. It must be noted though, that a ramp protocol is better suited to determine VT than LT, since the short increments of only one minute are not enough to reach a steady state blood lactate level, which may result in deviations from the real value. In addition, application of the ramp test with an increment of 25 W/min hindered us to exactly mirror the gas-exchange equilibrium between the production/transport site and exhalations for principal breath gases (e.g., O_2_ and CO_2_) to attain 95% or higher. As we continuously measured VOCs breath-by-breath to monitor progressive physio-metabolic changes under increasing power output until exhaustion, we could not address a steady-state of gas/VOC-exchange kinetics within the same setup. Nevertheless, our data shows, that this method is reproducible and yielded comparable results as in the previous study in an independent cohort. Acetone AT was only largely different from LT (48%) and VT (38%) in one participant of the study (volunteer 1). In this case acetone concentration barely increased during exercise, while the acetone concentration itself was unremarkable in comparison to the other participants and in the expected physiological range (286–316 ppbV). Besides the above-mentioned metabolic attributes, there are certain immediate physiological effects that further supports the behavior of acetone exhalation under exhaustive exercise. Sukul et al*.* demonstrated that breath acetone increases due to extra-alveolar exchange from the bronchial epithelium under higher respiratory flow and elevated minute ventilation (Sukul et al., 2016). Here, the increase in RR and VE until 90% of workload followed the bronchial contribution of VOCs like acetone (with high aqueous solubility and high blood gas partition coefficients).

Isoprene exhalation is well known to depend on physiology (King et al., 2009; Sukul et al., 2015) and consequently showed pronounced changes during the experiment and the strongest correlations to ventilatory parameters. Isoprene showed an immediate increase in the warm-up phase followed by a pronounced decrease during the ramp protocol to approximately 26% compared to the end of the warm-up period (Figure 3F). This behavior has been described before and may be attributed a wash-out from the working muscle compartment, which receives a high fraction of cardiac output and a metabolic activity change (King et al., 2010b). Alternatively, isoprene may be released from lipophilic storage sites through an increased diffusion (Miekisch et al., 2001; King et al., 2010b). With increasing workload exhaled isoprene concentrations decreased. Since isoprene exhalation depends on the ventilation-perfusion ratio, the pronounced increase of VE may surpass the effect of an increase in cardiac output (Schubert et al., 2012). There is evidence that sympathetic vasoconstriction takes place in skeletal muscles during exercise in order to balance the perfusion between active and inactive muscles (Buckwalter and Clifford, 2001; Joyner and Casey, 2015). This may result in a decreased transport of isoprene from the muscle compartments under higher workloads. The compartmental shift in perfusion under exercise is well reflected within the decreased transport of gut originated sulfides to lung.

Sulfur containing compounds methanethiol (Figure 3C) and dimethylsulfide (Figure 3E) both showed a significant decrease over the course of exercise, with a later onset of decrease for dimethylsulfide compared to methanethiol. Dimethylsulfide is produced in the gut as a breakdown product of methionine (Tangerman, 2009). During exercise blood shifts from the gastrointestinal tract to the muscle and lungs (Brouns and Beckers, 1993) which may lead to a decrease in bacterial activity and a decreased mixed venous concentration resulting in a decrease of alveolar dimethylsulfide (Szabó et al., 2015). While methanethiol may also be produced in the gut, transport from the blood into alveolar air is unlikely due to its high reactivity with blood which leads to an immediate irreversible binding and oxidation (Tangerman, 2002). Methanethiol is also known to be produced by oral bacteria in the mouth (Nakano et al., 2002). The decrease during exercise may be explained by a dilution effect from the increased ventilation. Additionally, changes in salivary composition (Chicharro et al., 1998) may influence its production and release.

### Compounds with a potentially mixed origin

Isopropanol can be endogenously formed from acetone *via* a nicotinamide adenine dinucleotide dependent redox-reaction (Jones and Rössner, 2007). While exhaled isopropanol tendentially showed a comparable trend to acetone (Supplementary Figure S1B), the changes were not statistically significant due to higher interindividual variation. This is no surprise since isopropanol can also originate from exogenous sources such as disinfectants and the production from isopropanol may strongly depend on diet and metabolic status (Pugliese et al., 2019).

While acrolein remained constant throughout the exercise (Supplementary Figure S1A), acetaldehyde (Figure 3B) and butanal (Figure 3G) showed a significant decrease in exhaled concentrations. Both acetaldehyde and butanal can have exogenous origin but may also be produced endogenously. Acetaldehyde is linked to ethanol metabolism (Umulis et al., 2005) and butanal may be produced by microbiota e.g. in the oral flora or may be linked to fatty acids metabolism. Both compounds may also be linked to oxidative stress (Orhan et al., 2006). Exercise is known to induce acute oxidative stress (Kawamura and Muraoka, 2018), which should result in an increase in aldehyde exhalation. In contrast to that, here aldehydes decreased with the very onset of workload. As the oxidative stress response to aerobic exercise is tissue/organ specific (Liu et al., 2000), irrespective of their production, the decrease in aldehydes may indicate higher oxidative stress in less perfused compartments than in active muscles. This is further supported by the significant negative correlation of these VOCs with HR. When the intensity of exercise supersedes moderate levels (>60–75% of VO_2_ max), the rate of fat oxidation is reduced (Coyle, 2007). This may partially explain a reduction of exhaled aldehyde concentrations.

C_4_H_8_O_2_ may refer to butyric acid among others. As can be seen in Table 2 and Supplementary Figure S1C it showed a comparable trend to acetone, but the changes did not reach statistical significance. In addition, an assignment to an individual substance is not straightforward. It may be interesting for future evaluations though, since it might have an endogenous or at least partially endogenous origin from the intestinal bacteria (Silva et al., 2020; Wang et al., 2020). Therefore, the decrease can be attributed to the same reasons explained for sulfur containing compounds.

### Exogenous compounds

Cyclohexadiene (C_6_H_8_) is a typical exogenous compound, nevertheless, it is noteworthy since, as a terpenoid, it showed a comparable trend to isoprene (Figure 3H). Acetonitrile, typically associated with cigarette smoke, showed a decreasing trend (Figure 3A).

Exhaustive exercise led to immediate and significant changes in exhaled VOC concentrations. Exhaled VOC profiling enabled real-time assessments of fast-occurring physio-metabolic changes and LT during exercise. We were able to successfully apply the method described by Schubert et al*.* (Schubert et al., 2012) for VOC based determination of AT in an independent cohort within a ramp exercise protocol underlining the validity of the approach in an independent setup. Sulfur containing compounds and butyric acid showed interesting trends potentially related to metabolic processes. Such compounds may be interesting for investigations of combined diet and exercise programs.

## Conclusion

Reproducible metabolic breath prints have offered non-invasive frontiers of applications to exercise physiology, physical (cardio-respiratory) fitness test, and sports medicine. Further cross-disciplinary studies in this direction may provide in-depth knowledge and understanding of the system wide communications between complex *in vivo* physiological, metabolic and/or biochemical processes and pathways. Simultaneous monitoring of oxidative stress, compartmental vasoconstriction, distribution/re-distribution of ventilation perfusion, broncho-pulmonary gas-exchange, microbial homeostasis and energy metabolism e.g. continuous glucose monitoring (CGM) may eventually lead us to the actual endogenous source(s) of VOCs i.e., an indispensable prerequisite for breath biomarker interpretation.

## Data Availability

The raw data supporting the conclusions of this article will be made available by the authors, without undue reservation.
