# Supplementary material for: Real-time metabolic monitoring under exhaustive exercise and evaluation of ventilatory threshold by breathomics: Independent validation of evidence and advances
Source: Front Physiol. 2022 Aug 12;13:946401. doi: 10.3389/fphys.2022.946401 (PMC9412033; doi:10.3389/fphys.2022.946401)
Supplement: Supplementary file 1 [file Table1.pdf]

Supplementary Material

Supplementary Figures and Tables

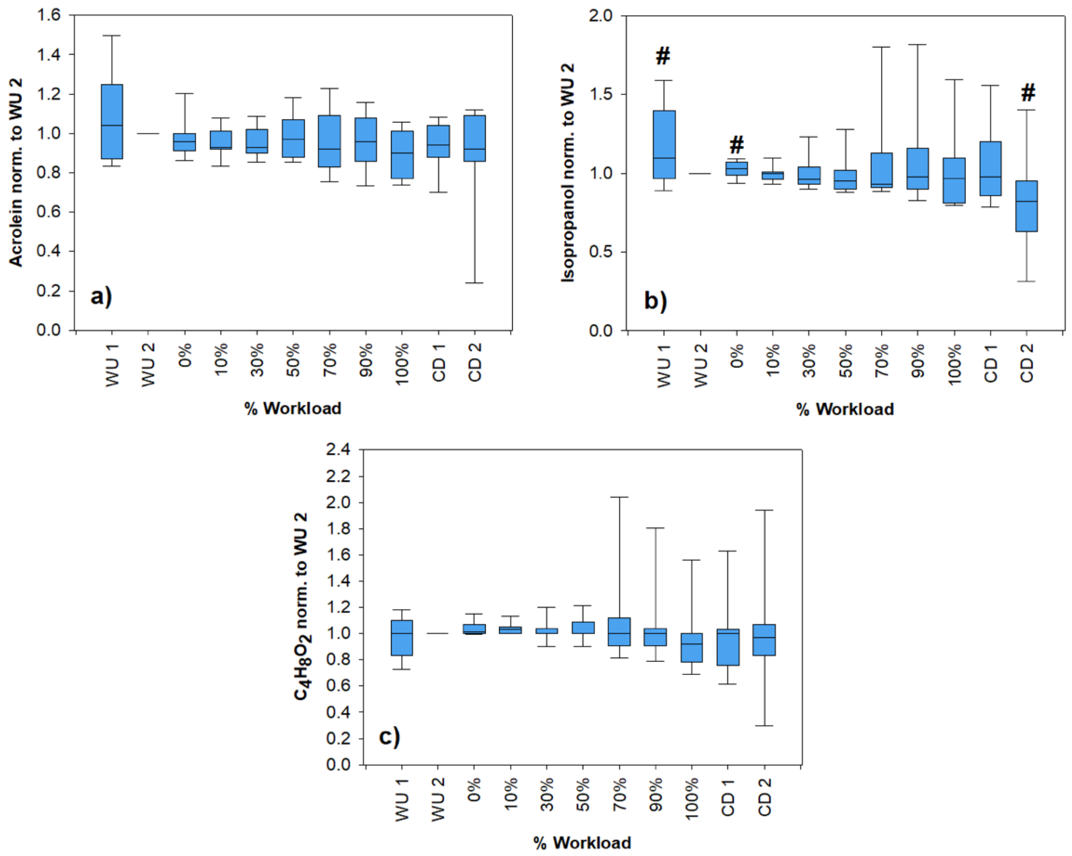

Supplementary Figure S1: Changes of acrolein(a), isopropanol (b) and $C_4H_8O_2$ (c) concentrations from all volunteers as a function of the relative workload. Absolute concentrations of all compounds were normalized to respective values at the last minute of warm-up (WU 2) for emphasis of relative changes. Statistically significant changes (Friedman repeated measures ANOVA on ranks, Student-Newman-Keuls post-hoc test, $p < 0.05$) compared to WU 2 are marked with a hash.

Supplementary Table S1: Statistical comparison within the first minute of warm up.

	***P*-value**
**Acetonitrile**	0.058*
**Acetaldehyde**	**0.002**
**Methanethiol**	**0.037**
**Acrolein**	0.218
**Acetone**	**0.009***
**Isopropanol**	**0.042**
**Dimethylsulfide**	**<0.001***
**Isoprene**	**<0.001**
**Butanal**	**0.002**
**Cyclohexadiene**	0.095
**$C_4H_8O_2$**	**0.013**

The mean over first three breaths of the minute were compared with the mean over the last three breaths of the minute from each volunteer. Wilcoxon signed rank sum tests (not-normally distributed data) or paired t-tests (normally distributed data) were performed to test statistical significance. $p<0.05$ was considered significant. Significant differences are marked in bold. Wilcoxon signed rank-sum tests are marked with an asterisk (*).

Supplementary Table S2: *P*-values from statistical comparison of spirometric parameters and HR at different time points with respect to WU 2.

	**WU 1**	**0%**	**10%**	**30%**	**50%**	**70%**	**90%**	**100%**	**CD 1**	**CD 2**
**Oxygen uptake**	**<0.001**	0.789	0.285	**<0.001**	**<0.001**	**<0.001**	**<0.001**	**<0.001**	**<0.001**	**<0.001**
**Carbon dioxide output**	**<0.001**	1.00	0.06	**<0.001**	**<0.001**	**<0.001**	**<0.001**	**<0.001**	**<0.001**	**<0.001**
**RER**	0.082	0.142	**0.007**	**0.014**	**<0.001**	**<0.001**	**<0.001**	**<0.001**	**<0.001**	**<0.001**
**Minute ventilation**	**<0.001**	0.894	**0.037**	**<0.001**	**<0.001**	**<0.001**	**<0.001**	**<0.001**	**<0.001**	**<0.001**
**Tidal volume**	**<0.001**	0.756	0.57	**0.003**	**<0.001**	**<0.001**	**<0.001**	**<0.001**	**<0.001**	**0.017**
**Respiratory rate**	0.227	0.593	**0.037**	0.251	**<0.001**	**<0.001**	**<0.001**	**<0.001**	**<0.001**	**<0.001**
**Heartrate**	1.00	**0.022**	**0.007**	**<0.001**	**<0.001**	**<0.001**	**<0.001**	**<0.001**	**<0.001**	**<0.001**

Friedman repeated measures ANOVA on ranks in combination with Student-Newman-Keuls post-hoc was performed with the exception of TV. Due to normal distribution, a one-way repeated measures ANOVA was performed in this case in combination with Student-Newman-Keuls post-hoc test. $p<0.05$ was considered significant. Significant differences are marked in bold.

Supplementary Table S3: *P*-values from comparison of exhaled VOC concentrations at different time points with respect to WU 2.

	WU 1	0%	10%	30%	50%	70%	90%	100%	CD 1	CD 2
**Acetonitrile**	**<0.001**	1.00	**0.005**	**<0.001**	**<0.001**	**<0.001**	**<0.001**	**<0.001**	**<0.001**	**<0.001**
**Acetaldehyde**	**0.005**	0.405	**0.006**	**<0.001**	**<0.001**	**<0.001**	**<0.001**	**<0.001**	**<0.001**	**<0.001**
**Methanethiol**	**<0.001**	**0.027**	**0.017**	**0.003**	**<0.001**	**<0.001**	**<0.001**	**<0.001**	**<0.001**	**<0.001**
**Acrolein**	0.119	0.119	0.119	0.119	0.119	0.119	0.119	0.119	0.119	0.119
**Acetone**	**0.001**	0.122	0.267	**0.002**	**<0.001**	**<0.001**	**<0.001**	**0.579**	**0.001**	**0.262**
**Isopropanol**	**0.035**	0.032	0.826	0.579	0.799	0.122	0.782	0.152	0.489	**0.01**
**Dimethylsulfide**	0.488	0.589	0.332	0.527	0.299	**<0.001**	**<0.001**	**<0.001**	**<0.001**	**<0.001**
**Isoprene**	**<0.001**	0.579	0.079	**<0.001**	**<0.001**	**<0.001**	**<0.001**	**<0.001**	**<0.001**	**<0.001**
**Butanal**	**<0.001**	**<0.001**	0.89	**<0.001**	**<0.001**	**<0.001**	**<0.001**	**<0.001**	**<0.001**	**<0.001**
**Cyclohexadiene**	0.405	0.782	0.122	0.228	**0.017**	**<0.001**	**<0.001**	**<0.001**	**<0.001**	**0.007**
**$C_4H_8O_2$**	0.063	0.063	0.063	0.063	0.063	0.063	0.063	0.063	0.063	0.063

Friedman repeated measures ANOVA on ranks in combination with Student-Newman-Keuls post-hoc was performed. $p<0.05$ was considered significant. Significant differences are marked in bold.
